# Are the Self-esteem, Self-efficacy, and Interpersonal Interaction of Junior College Students Related to the Solitude Capacity?

**DOI:** 10.3390/ijerph17218274

**Published:** 2020-11-09

**Authors:** Shang-Yu Yang, Shih-Hau Fu, Po-Yu Wang, Ying-Lien Lin, Pin-Hsuan Lin

**Affiliations:** 1 Department of Healthcare Administration, College of Medical and Health Science, Asia University, Taichung 413, Taiwan; henry879019@yahoo.com.tw; 2 Department of Acupressure Technology, Chung Hwa University of Medical Technology, Tainan 71703, Taiwan; ptchrisman@gmail.com; 3 Department of Pediatric Emergency, Changhua Christian Children Hospital, Changhua 500, Taiwan; dama0115@yahoo.com.tw; 4 Department of Industrial and Information Management, National Cheng Kung University, Tainan 701, Taiwan; t10025023@gm2.nutn.edu.tw; 5 Department of Health and Beauty, Shu Zen Junior College of Medicine and Management, Kaohsiung 821, Taiwan

**Keywords:** self-esteem, self-efficacy, interpersonal relationship, solitude capacity

## Abstract

*Background:* Studies on the solitude capacity of university students have been extremely limited and failed to clearly illustrate the correlation of solitude capacity with internal psychological variables and the favorability of interpersonal relationships. The aim of this study was to explore the correlation of college students’ solitude capacity with scores for self-esteem, self-efficacy, and interpersonal relationships. *Method:* A cross-sectional study was adopted for this study. Data were collected from a university in southern Taiwan using a structured questionnaire, the content of which included demographic data and scores from the Rosenberg Self-Esteem Scale (RSE), the General Self-Efficacy Scale (GSE), the Interpersonal Relationship Scale (IRS), and the Solitude Capacity Scale (SCS). *Results:* The final sample comprised 562 participants (mean age = 17.51 ± 1.27 years). Adjustment of the demographic variables yielded a significantly positive correlation in the total RSE and SCS (*p* < 0.01) scores and that in the total GSE and SCS (*p* < 0.01) scores. Moreover, the relationship with family (IRS subscale) and total SCS score (*p* < 0.05) exhibited a significant positive correlation. *Conclusion:* The findings of this study reveal that solitude capacity is significantly correlated with self-esteem, self-efficacy, and the favorability of family relationships.

## 1. Introduction

According to Sullivan’s interpersonal theory [1] and Bowlby’s attachment theory [2], during their time as college students, individuals pursue interpersonal interaction. However, if they seek solitude, this may reflect psychological distress and social difficulties (e.g., rejection by peers) that lead to voluntary solitude [3]. Solitude can relieve negative emotions and stress; however, a vicious cycle may occur if an individual retreats from unpleasant situations. This form of solitude is dangerous and may lead to numerous mental illnesses [4,5]. Despite this, solitude also has constructive functions. Solitude during adolescence provides a space for contemplation and silence, reducing the pressure from overemphasis on peer relationships; moreover, solitude during early adulthood can facilitate reflection on financial and work-related pressure [6]. Furthermore, individuals with solitude capacity exhibit healthier interpersonal relationships and better well-being, and appropriate solitude contributes to physical and mental health [4,7], enhances creativity [8], and assists self-healing [9]. During the stage of university studies, although individuals tend to feel lonely when they are away from their peers (due to their desire for peer recognition and interpersonal relationships), they also require solitude to explore and establish their self-identity [10]. The more comfortable an individual feels when he or she is alone, the more positive an individual’s solitude experience is, and the more opportunity he or she has to acquire the constructive function of solitude; accordingly, solitude capacity is a major factor in individuals’ positive solitude experiences [11]. Therefore, understanding university students’ solitude capacity is a topic meriting discussion.

Many studies have noted that internal psychological variables are related to preference for solitude. For example, adolescents with low self-esteem and low self-efficacy prefer to solitude, which is probably because of the social and emotional disorders in their development [3,12,13]. Because adolescents with low self-esteem are not easily accepted by their classmates in school, they are more likely to be separated from their peers and spend more time alone. Besides, adolescents with low self-efficacy may have lower motivation to interact with their classmates and tend to be alone [14,15]. Although adolescents with low self-esteem or low self-efficacy prefer to be alone, they tend to have negative solitude experiences such as loneliness or anxiety, which are not conducive to the development of the solitude capacity [16]. Although the correlations involving adolescents’ internal psychological variables and solitude preferences have been discussed, few studies have explored solitude capacity in depth, which is different from solitude preference [7]. Solitude preference refers to active (or forced) experience of a solitary environment; solitude capacity describes ability to use solitary time constructively and enjoy solitude; thus, the two should not be confused [7,11]. However, the correlation between an individual’s internal psychological variables (including self-esteem and self-efficacy) and solitude capacity remains unclear.

Although every individual requires solitude, human interaction is also required, particularly by college students [1]. The definition of solitude describes separation from others in on an emotional or psychological level rather than physically. Solitude therefore does not necessarily describe an individual’s physical isolation [5,11]. However, studies on solitude have focused on solitude preference, regarding individuals as persons who like to be alone in a context; thus, most findings have indicated that individuals with relatively few interpersonal relationships or people who avoid interpersonal relationships demonstrate a preference for solitude [3,17]. In fact, individuals with high capacity for solitude tend to possess relatively constructive personality traits, such as creativity, intimacy, and superior adaptability to interpersonal relationships [4,16]. However, the aforementioned personality traits with solitude capacity all ostensibly benefit interpersonal relationships and interpersonal interaction; accordingly, the relationship between interpersonal relationships and solitude capacity requires further research for verification.

In addition, adolescents’ solitude is highly correlated with loneliness. In other words, individuals who are prone to feeling lonely are more likely to be alone [18]. Barreto, et al. [19] reported that males are more likely to feel lonely than females, and younger people are more likely to feel lonely than the middle-aged. Nevertheless, Öner and Arslantaş’s study [20], which reported that females were more likely to feel lonely than males. Pyle and Evans [21] showed that feeling of belonging in a group or living with family or friends is less likely to feel lonely. Moreover, an individual with less exercise frequency [22] or less disposable money [23] is more likely to feel lonely. Thus, when investigating adolescents’ solitude capacity, some individual characteristics that may influence the state of solitude should be taken into account.

According to Erikson’s psychosocial developmental theory, the university period is an important stage for the development of individual self-identity [24]. At university, individuals are more capable of self-awareness and emotional reflection if they possess solitude capacity [25]. Solitude capacity is the ability to enjoy one’s own company and regulate one’s own emotions, which helps in the building of one’s personality structure and development of self-identity [4,16,25]; thus, the value of solitude cannot be overlooked. However, the scarce research on university students’ solitude capacity means that a clear explanation of the correlation of scores related to internal psychological variables (self-esteem and self-efficacy), interpersonal relationships, and solitude capacity is impossible. Accordingly, this study explored the correlation of solitude capacity with the favorability of university students’ self-esteem, self-efficacy, and interpersonal relationships. Through understanding the respective correlation, relevant information can be provided for education departments and parents to reduce risks for adolescents’ health. Based on the results of our literature review, we propose three hypotheses: compared with adolescents with relatively poor solitude capacity, those with better self-esteem (Hypothesis 1), self-efficacy (Hypothesis 2), and interpersonal interaction (Hypothesis 3) would exhibit better solitude capacity.

## 2. Methods

### 2.1. Study Design and Subject Recruitment

A cross-sectional study design was adopted. This study used a structured questionnaire to recruit participants from a college in southern Taiwan. The questionnaire collection period was from 1 May to 31 July 2019, a total of 3 months. First, the research assistant attended each class to explain the research content and recruited students. After written consent was obtained from participants, they were taken to a classroom to answer the questionnaires. Students aged 18 years or younger required the consent of their guardians. In addition, the inclusion criteria were as follows: (1) the ability to complete the questionnaire and (2) the ability to communicate in Mandarin or Taiwanese and to understand questionnaire content. Individuals diagnosed by doctors with mental illness or relevant symptoms were excluded. Ethical approval for the study was obtained from the National Cheng Kung University Human Research Ethics Committee (NCKU HREC-E-108-032-2). Ultimately, 564 students agreed to participate in this study and returned consent forms. However, 2 questionnaires were discovered to be incomplete after review; thus, 562 complete questionnaires were finally collected.

### 2.2. Questionnaire

The questionnaire in this study consisted of five sections. The first involved basic demographic data, including sex, age, body mass index, religion, number of days of exercise per week (each exercise session must last more than 30 min), monthly allowance, relationship status, and type of residence (university dormitory, off-campus dormitory, or family home). The second section featured the Rosenberg Self-Esteem Scale (RSE), which was used to measure self-esteem. The RSE, designed by Morris Rosenberg [26], is a self-evaluation scale and contains 10 items. A 4-point Likert-like scale is used for scoring, with a score of 1–4 assigned according to participants’ responses of strongly disagree, disagree, agree, and strongly agree, respectively. The total score ranges from 10 to 40, with a higher total score indicating higher self-esteem. The RSE possesses favorable reliability and validity [26]. The Cronbach’s alpha of the total RSE score of this study was 0.85.

In the third section, the General Self-Efficacy Scale (GSE) was used to measure self-efficacy. Designed by Schwarzer and Jerusalem (1995), the GSE is a self-evaluation scale with 10 items [27]. A 4-point Likert scale was adopted for scoring, with scores from 1 to 4 assigned according to participants’ responses of strongly disagree, disagree, agree, and strongly agree, respectively. The total score ranges from 10 to 40, and a higher total score indicates more favorable self-efficacy. The GSE possesses favorable reliability and validity [28]. The Cronbach’s alpha of the total GSE score of this study was 0.96.

The fourth section was based on the Interpersonal Relationship Scale for College Students (IRS), used to measure interpersonal relationships. The IRS, designed by Su [29], is a self-evaluation scale with 25 items, which are divided into five dimensions: social acceptance (4 items), family relationships (6 items), sense of humor (5 items), peer relationships (5 items), and intimate friendship (5 items). A 4-point Likert scale is adopted for scoring, in which a score of 1–4 is assigned according to the participants’ responses of very inconsistent, not very consistent, mostly consistent, and very consistent, respectively. The total score ranges from 25 to 100, and a higher total score indicates more favorable interpersonal relationships. The IRS possesses favorable reliability and validity [29]. The Cronbach’s alpha of the total IRS score and its five dimensions (social acceptance, family relationships, sense of humor, peer relationships, and intimate friendship) for this study were 0.94, 0.85, 0.87, 0.79, 0.74, and 0.89, respectively.

The fifth section was based on the Solitude Capacity Scale (SCS), used to measure solitude capacity. The SCS was translated by Wu and Chen [30] based on the Capacity to be Alone Scale by Larson and Lee [11]. It is a self-evaluation scale and contains two dimensions: solitary coping (10 items) and solitary comfort (10 items), a total of 20 items. Solitary coping refers to an individual’s ability to use time alone constructively for contemplation and self-reflection, whereas solitary comfort describes whether an individual is happy alone. A 4-point Likert scale is adopted for scoring, in which scores of 1 to 4 are assigned according to the participants’ responses of very inconsistent, inconsistent, consistent, and very consistent, respectively. The total scores range from 20 to 80, and a higher total score (subscale score) indicates more favorable solitude capacity. The SCS possesses favorable reliability and validity [30]. The Cronbach’s alpha of the total SCS score and its two dimensions (solitary coping and solitary comfort) of this study were 0.86, 0.78, and 0.78, respectively.

### 2.3. Statistical Analysis

This study employed SPSS 22.0 (IBM Corp., Armonk, NY, USA) for mac for data analysis. First, the demographic data, RSE, GSE, IRS, and SCS were presented using descriptive statistics. Next, the correlations among RSE, GSE, IRS, and SCS were explored using Pearson correlation coefficient analysis. Finally, the aforementioned correlations were tested using multiple regression analysis. The total score for the SCS and its 2 subscale scores were input into the multiple regression model as dependent variables, after which the total scores for the RSE, GSE, and IRS (and the IRS subscale scores) were input into the model as independent variables; moreover, the demographic variables were adjusted. Studies have indicated that, among the demographic variables, sex [31], age [32], body mass index [33], religion [34], number of days of exercise per week [32], monthly allowance [35], relationship status [36], and type of residence [32] may affect the independent and dependent variables in this study. In addition, each multiple regression model was subjected to collinearity diagnosis, and the variance inflation factor values of the independent variables in all the models were less than 10; thus, collinearity was not a problem [37].

## 3. Results

### 3.1. Participant Demographics

This study ultimately recruited 267 male students and 295 female students, with an average age of 17.51 ± 1.27 years (age range = 16–19 years old). Table 1 shows the students’ demographic data. More than half of the students had no religion (57.5%), exercised fewer than two days per week (52.7%), were single (64.9%), and lived at home (67.8%); moreover, most students (45.4%) received a weekly allowance of less than 4000 NTD (New Taiwan Dollars). The students’ total scores for the RSE, GSE, and IRS were 27.91 ± 5.00, 25.12 ± 7.11, and 80.89 ± 12.44, respectively. Their scores for the five dimensions of the IRS (social acceptance, family relationships, sense of humor, peer relationships, and intimate friendship) were 16.17 ± 2.95, 19.93 ± 3.79, 14.81 ± 2.91, 13.17 ± 2.20, and 16.81 ± 2.94, respectively. In addition, their total score for the SCS was 56.54 ± 8.43, whereas their scores for the solitary coping subscale and solitary comfort subscale were 28.63 ± 4.63 and 27.92 ± 4.56, respectively.

### 3.2. Pearson Correlation Coefficient Analysis

Table 2 shows the results for the correlations of the RSE, GSE, IRS, and SCS sores analyzed using the Pearson correlation coefficient. The total SCS score had a significant positive correlation with the total RSE score (r = 0.18, *p* < 0.01), the total GSE score (r = 0.16, *p* < 0.01), and the IRS subscale of relationship with family (r = 0.11, *p* < 0.01). The SCS subscale, the solitary coping subscale shared a significant positive correlation with the GSE total score (r = 0.14, *p* < 0.01), whereas the solitary comfort subscale had a significant positive correlation with the total RSE score (r = 0.26, *p* < 0.01), total GSE score (r = 0.15, *p* < 0.01), total IRS score (r = 0.09, *p* < 0.05), and IRS subscale for relationship with family (r = 0.14, *p* < 0.01).

### 3.3. Multiple Regression Analysis

Table 3 shows the analysis results for the respective correlation of total RSE score, total GSE score, and total IRS score (and IRS subscale scores) with the total SCS score subject to the adjusted demographic variables in the multiple regression model. The total scores for the RSE and SCS (R^2^: 0.05, B: 0.28, SE: 0.07, *p* < 0.01) exhibited a significant positive correlation, indicating that higher self-esteem results in more favorable solitude capacity. The total GSE total score and total SCS score (R^2^: 0.05, B: 0.20, SE: 0.05, *p* < 0.01) shared a significant positive correlation, meaning that increased self-efficacy leads to more favorable solitude capacity. In addition, the relationship with family subscale (B: 0.27, SE: 0.13, *p* < 0.05) was significantly positively correlated with the total SCS score, indicating that a more favorable relationship with family leads to more satisfactory solitude capacity.

Table 4 shows the analysis results for the respective correlation of total RSE score, total GSE score, and total IRS score (and IRS subscale scores) with the SCS subscales (solitary coping subscale and solitary comfort subscale). The solitary coping subscale was significantly positively correlated with the total GSE score (R^2^: 0.04, B: 0.10, SE: 0.03, *p* < 0.01), meaning that increased self-efficacy leads to increased solitary coping capacity. The solitary comfort subscale exhibited a significant positive correlation with the total RSE score (R^2^: 0.09, B: 0.22, SE: 0.04, *p* < 0.01), the total GSE score (R^2^: 0.06, B: 0.10, SE: 0.03, *p* < 0.01), and the relationship with family subscale (B: 0.19, SE: 0.07, *p* < 0.01), which means that increased self-esteem, increased self-efficacy, and more favorable relationships with family members result in an increased level of solitary comfort.

## 4. Discussion

This is one of few studies exploring university students’ solitude capacity. With adjustment for demographic variables, the findings of this study indicate that university students’ self-esteem, self-efficacy, and relationship with family are significantly positively correlated with solitude capacity. Therefore, the results were in support of our Hypotheses 1 to 3. Furthermore, the results reveal that greater self-efficacy may lead to improved solitary coping, whereas improved self-esteem, self-efficacy, and relationship with family result in a higher level of solitary comfort. The regression model in this study exhibited limited explanatory power, but the results contradicted those of other studies on solitude preference [3,12,13,17]. Other research noted potential problems such as low self-esteem, low self-efficacy, and poor interpersonal relationships among university students with solitude preference. In terms of solitude capacity, the present study showed that university students with solitude capacity exhibited more favorable self-esteem, self-efficacy, and family relationships. Our findings, therefore, provided further insights into the role of solitude among university students. However, the design of this research precludes determination of the causal relationship; therefore, the causal relationship between various variables requires further investigation.

Self-esteem is a major factor influencing adolescent lives [38]. The results of this study indicate that students with low self-esteem may have poor solitude capacity and are particularly prone to discomfort during solitude. As such, individuals with low self-esteem who in solitude experience more difficulty in constructive thinking and activities, and are prone to negative emotions such as anger, stress, and aimlessness. Moreover, studies have noted a close correlation between self-esteem and loneliness—individuals with low self-esteem are more likely to experience loneliness in solitude, which, in turn, reduces their positive solitude capacity [39,40,41,42]. Self-esteem describes an individual’s self-evaluation and feelings regarding himself or herself [26]. Individuals with low self-esteem usually lack confidence and tend to make negative self-evaluations regarding their social behavior, causing them to avoid social environments and, in turn, increasing their loneliness [40]. This loneliness is caused not only by inadequate interaction with a group of friends but also by a lack of intimate friendship. Thus, McWhirter et al. [38] proposed that enhancing an individual’s self-esteem should be considered one approach for reducing loneliness. In addition, more external social support can elevate an individual’s self-esteem [43]; therefore, university students are advised to learn social skills and to increase social interaction and support through participation in social services, thereby improving their self-esteem and mental health [44].

Self-efficacy plays a crucial role in social behaviors [45]. An improvement in self-efficacy assists with the mediation of conflicts and stress involved in social behaviors [46] and the enhancement of interpersonal interaction [32]. On-campus social behaviors greatly affect university students. Favorable social behaviors are conducive to approval from peers and teachers, which can produce positive development for students’ own learning [47]. Self-efficacy also has a major role in stress management [48]. In the same stressful situation, individuals with high levels of self-efficacy are better equipped to endure and overcome pressure [49]. Furthermore, greater self-efficacy indicates that an individual has a greater belief in his or her capability to manage various resources required for a specific task [50]. Therefore, university students with greater self-efficacy may possess more favorable social behaviors, stress coping, and confidence in achieving their goals. In other words, university students with high levels of self-efficacy may not have chosen solitude due to unsatisfactory social interactions. They have the capacity to adjust to the pressures of solitude (e.g., loneliness) and are more able to use their solitude to contemplate obligations. This also explains the findings of this study, namely, the correlation between self-efficacy and solitude capacity (solitary coping and solitary comfort).

Studies have indicated that a high level of solitude preference may be due to poor interpersonal relationships, caused by the inability to establish favorable interpersonal relationships within a group [3,17]. By contrast, this study discovered that students with worse interpersonal relationships possess worse solitude capacity; that is, poor interpersonal relationships may cause a preference for solitude, but this is accompanied by poor solitude capacity, which may negatively affect an individual’s mental health. Furthermore, the regression model showed that students exhibiting more favorable relationships with their family also possess more satisfactory solitude capacity (solitary comfort). Family support (e.g., the company of and assistance from family members) facilitates development of independence during adolescents’ growth [51]. In addition, favorable family relationships benefit adolescents’ emotional regulation during their growth and positively affect the development of their self-esteem and interpersonal relationships [52,53]. The findings of this study also suggest that self-esteem and interpersonal relationships contribute to solitude capacity, which may explain the correlation between relationship with family and solitude capacity noted in this study.

Solitude, which most students experience [54], does not always produce only negative feelings, which are only one possible effect of solitude. It provides individuals with the opportunity to listen to their inner feelings and emotions, enabling suppressed emotions to be released and vented, providing the opportunity for re-examination or analysis of a predicament from an alternative perspective [25]. During adolescence, solitude provides the opportunity for self-reflection and self-exile, and students learn to gain positive solitude experience in the process of being alone; in adulthood, people reflect on financial and professional pressure in solitude [6,25]. The development of solitary capacity among college students is beneficial to both their physical and mental health [7].

Several limitations should be considered in interpreting the results of this study. The most notable one is the low explanatory power of the regression model. However, despite the limited explanatory power of the model, the findings of this study provide a valuable reference for the study of university students’ solitude capacity. The second limitation is that the measurements of the scales in this study are self-evaluated. Although these scales have been widely used in various studies and provide favorable psychological measurements, they nonetheless do not provide a full representation of personality and behavioral performance. Third, because the students recruited in this study are all from the same university, and because personality and interpersonal relationships undergo changes and transformation during university, the explanatory power of the model in this study is limited. Moreover, this study may not be applicable to western countries where the upbringing culture is different. Fourth, this study is exploratory research on the relationship between university students’ solitude capacity, personality traits, and interpersonal relationships. Although the participants’ sexes were separately analyzed in the regression analysis in this study, the results were similar to those from the analysis in which the sexes were combined; thus, sex-segregated analysis was not performed in this study. However, considering that the variables in this study may nonetheless have been affected by sex-related differences, further analysis of differences between the sexes is recommended. Fifth, despite the availability of information on demographic variables that may potentially affect the participants’ independent and dependent variables, no data related to the students’ families or academic performance were obtained, which may also have influenced the explanatory power of our model. Thus, the inclusion of family variables or academic performance status is recommended for future studies (e.g., family’s financial status or academic score). It is also suggested that future research can further explore the relationship between other demographic variables and solitude capacity. Finally, this study used cross-sectional research and thus could not explain causal relationships. Despite the aforementioned limitations, the study provided findings related to the correlation of university students’ solitude capacity with personality traits and interpersonal relationships. The results will improve understanding of adolescents’ solitude capacity in relevant institutions or professions.

## 5. Conclusions

The findings of this study suggested that self-esteem, self-efficacy, and relationship with family exhibit a significant positive relationship with solitude capacity. Therefore, university students are advised to employ solitude to clear their thoughts, which not only increases their thoughtfulness but also facilitates positive personality development.

## Figures and Tables

**Table 1:** Demography of the participants.

Demographic Variable	Total
	*n* = 562
Sex	
Male	267 (47.5%)
Female	295 (52.5%)
Age (mean ± SD)	17.51 ± 1.27
BMI (mean ± SD)	20.68 ± 3.66
Religion (*n*, %)	
No	323 (57.5%)
Yes	239 (42.5%)
Exercise per week	
0–1 days	296 (52.7%)
2–3 days	188 (33.5%)
≥4 days	78 (13.9%)
Monthly allowance	
< 4000 NTD	255 (45.4%)
4000–5999 NTD	136 (24.2%)
6000–7999 NTD	54 (9.6%)
≥8000 NTD	117 (20.8%)
Relationship status (have a boy/girl friend)	
No	365 (64.9%)
Yes	197 (35.1%)
Type of residence	
Home	381 (67.8%)
School dormitory	54 (9.6%)
Off-campus rental house	127 (22.6%)

NTD: New Taiwan Dollars; SD: standard deviation; BMI: body mass index.

**Table 2:** Correlation coefficients among the Solitude Capacity Scale, Rosenberg Self-Esteem Scale, General Self-Efficacy Scale, and Interpersonal Relationship Scale for College Students with subscales.

Variable	Total Score	Solitary Coping (Subscale)	Solitary Comfort (Subscale)
RSE	0.18 **	0.07	0.26 **
GSE	0.16 **	0.14 **	0.15 **
IRS			
Total score	0.07	0.05	0.09 *
Social acceptance	0.04	0.03	0.05
Relationship with family	0.11 **	0.07	0.14 **
Sense of humor	0.05	0.04	0.05
Peer relationship	0.06	0.04	0.07
Close friendship	0.03	0.02	0.04

* *p* < 0.05, ** *p* < 0.01. RSE: Rosenberg Self-Esteem Scale; GSE: General Self-Efficacy Scale; IRS: Interpersonal Relationship Scale for College Students.

**Table 3:** Multiple regression analysis for identifying Solitude Capacity Scale ^†^ with total score significantly related to Rosenberg Self-Esteem Scale, General Self-Efficacy Scale, and Interpersonal Relationship Scale for College Students with subscales.

	Dependent Variables	Solitude Capacity Scale (Total Score)							
Independent Variables		R^2^	Adjusted R^2^	F	B	SE	Beta	95% CI	*p*
RSE total score		0.05	0.04	3.01	0.28	0.07	0.16	0.14, 0.42	<0.01 **
GSE total score		0.05	0.04	3.06	0.20	0.05	0.16	0.10, 0.29	<0.01 **
IRS total score		0.03	0.01	1.74	0.04	0.03	0.06	−0.01, 0.10	0.13
IRS subscales		0.04	0.01	1.56					
Social acceptance					−0.05	0.26	−0.02	−0.56, 0.45	0.84
Relationship with family					0.27	0.13	0.12	0.02, 0.52	0.03 *
Sense of humor					0.14	0.19	0.05	−0.23, 0.52	0.45
Peer relationship					0.18	0.28	0.05	−0.38, 0.74	0.53
Close friendship					−0.28	0.26	−0.10	−0.80, 0.24	0.29

^†^ Adjusted for sex, age, BMI, religion, exercise per week, monthly allowance, relationship status, and living place; * *p* < 0.05, ** *p* < 0.01. CI: confidence interval; S.E.: standard error; B: regression coefficient; IRS: Interpersonal Relationship Scale for College Students; GSE: General Self-Efficacy Scale; RSE: Rosenberg Self-Esteem Scale.

**Table 4:** Multiple regression analysis for identifying Solitude Capacity Scale ^†^ with two dimensions significantly related to Rosenberg Self-Esteem Scale, General Self-Efficacy Scale, and Interpersonal Relationship Scale for College Students with subscales.

	Dependent Variables		Solitary Coping Subscale							Solitary Comfort Subscale							
Independent Variables		R^2^	Adjusted R^2^	F	B	SE	Beta	95% CI	*p*	R^2^	Adjusted R^2^	F	B	SE	Beta	95% CI	*p*
RSE total score		0.02	0.01	1.29	0.06	0.04	0.07	−0.02, 0.14	0.14	0.09	0.08	6.12	0.22	0.04	0.24	0.14, 0.29	<0.01 **
GSE total score		0.04	0.02	2.48	0.10	0.03	0.15	0.04, 0.15	<0.01 **	0.06	0.04	3.84	0.10	0.03	0.15	0.05, 0.15	<0.01 **
IRS total score		0.02	0.00	1.16	0.02	0.02	0.04	−0.02, 0.05	0.31	0.04	0.03	2.71	0.03	0.02	0.08	−0.00, 0.06	0.07
IRS subscales		0.02	0.00	0.95						0.05	0.03	2.42					
Social acceptance					−0.03	0.14	−0.02	−0.31, 0.25	0.85				−0.00	0.14	−0.00	−0.27, 0.27	0.98
Relationship with family					0.08	0.07	0.07	−0.06, 0.22	0.24				0.19	0.07	0.15	0.06, 0.32	<0.01 **
Sense of humor					0.11	0.11	0.07	−0.10, 0.31	0.31				0.03	0.10	0.02	−0.17, 0.23	0.77
Peer relationship					0.05	0.16	0.02	−0.26, 0.36	0.75				0.11	0.15	0.05	−0.19, 0.41	0.47
Close friendship					−0.11	0.15	−0.07	−0.40, 0.18	0.44				−0.17	0.14	−0.11	−0.45, 0.11	0.22

^†^ Adjusted for sex, age, BMI, religion, exercise per week, monthly allowance, relationship status, and living place; ** *p* < 0.01; CI: confidence interval; S.E.: standard error; B: regression coefficient; IRS: Interpersonal Relationship Scale for College Students; GSE: General Self-Efficacy Scale; RSE: Rosenberg Self-Esteem Scale.
